# The Neurotropic Activity of Novel Dermorphin Analogs Active in Systemic and Noninvasive Administration

**DOI:** 10.3390/ijms26178437

**Published:** 2025-08-29

**Authors:** Vladislav Deigin, Nikolay Korobov, Olga Volpina, Natalia Linkova, Anastasiia Diatlova, Dmitrii Medvedev, Alexander Krasichkov, Victoria Polyakova

**Affiliations:** 1 Shemyakin-Ovchinnikov Institute of Bioorganic Chemistry, Russian Academy of Sciences, Miklukho-Maklaya St., 16/10, 117997 Moscow, Russia; vdeigin@gmail.com (V.D.); volpina@ibch.ru (O.V.); me@diatlova.ru (A.D.); 2 Department of Pharmacology, Faculty of Fundamental Medicine, Lomonosov Moscow State University, GSP-1, Leninskie Gory, 119991 Moscow, Russia; nvkorobov@mail.ru; 3 Departments of Hospital Surgery, Belgorod National Research University, 308015 Belgorod, Russia; vopol@yandex.ru; 4 The Department of Social Rehabilitation and Occupational Therapy of the St. Petersburg Medical and Social Institute, 72A Kondratievsky St., 195271 St. Petersburg, Russia; rsc-ide@yandex.ru; 5 Department of Radio Engineering Systems of Electrotechnical University LETI, 5F Prof. Popova Str., 197022 St. Petersburg, Russia; krass33@mail.ru

**Keywords:** neurotrophic peptides, diketopiperazine, analgetic activity, opioid activity, intranasal administration, intragastric administration

## Abstract

The neuropeptide’s multifaceted involvement in various components of neural homeostasis impacts pain and behavioral regulation. Dermorphin is a highly potent neuropeptide, extracted from the skin of the Amazon frog (*Phyllomedusa sauvagei*). The unique feature of dermorphin is the D-Ala residue in its sequence, which has inspired researchers to search for dermorphin analogs for use as pharmaceuticals. The primary objective of this study was to synthesize several new linear and cyclic dermorphin analogs and evaluate them as potential non-invasive analgesics. Using our method for converting linear peptides into 2,5-diketopiperazine(2,5-DKP) derivatives, which stabilize peptide structures, we synthesized several new dermorphin linear peptides and chimeric cyclopeptidomimetics. These compounds were tested in vitro and in vivo to determine their biological activities and potential applicability as pharmaceuticals. The “guinea pig ileum” (GPI) test was used for the evaluation of in vitro opioid activity. D2 showed the highest activity, and cyclopeptides D3 and D4 showed high activity. We can assume that the dermorphin analogs D2, D3, and D4 are potent agonists of µ-type opioid receptors and have high opioid activity. However, this needs to be verified using molecular modeling methods in further research. The analgesic effects of dermorphins were evaluated in the “hot-plate” and “tail-flick” tests. In rats, D2 dermorphin analogs demonstrated a dose-dependent analgesic effect in the “Water Tail-Flick” test after intranasal administration. A smaller dose of 0.5 µg/kg resulted in 40% analgesia and a short-term state of stupor. The maximum long-lasting analgesia was observed at a dose of 1.0 µg/kg, which induced complete stupor. The analgesic effect of peptide D2 after intraperitoneal administration at a 5.0 mg/kg dose was over 50%. The “open-field” test demonstrated a dose-dependent (15, 50, 150 μg/kg) peptide D2 suppression effect on behavioural reactions in rats following intranasal administration. A new modification of linear peptides, combined with a 2,5-DKP scaffold (D3 and D4), proved promising for oral use based on the results of analgesic effect evaluation in mice following intragastric administration.

## 1. Introduction

Neuropeptides, a diverse family of biologically active peptides, play a crucial role in neuroregulation and the management of pain. Their intricate involvement in multiple components of neural homeostasis, including sensory perception and behavioral regulation, underscores their significance. In this context, dermorphin—a unique neuropeptide—stands out due to its exceptional properties and potential applications in pain management and neuroscience [1].

The Dermorphin family comprises seven endogenous molecules, represented by exclusive new eukaryotic regulatory peptides that contain a D-amino acid residue in their sequence. In contrast, their precursor, prodermorphin, has the L-isomer of alanine (Ala) in the same position. This transformation occurs through post-translational modifications, resulting in the conversion of L-Ala to D-Ala. The unique ability to incorporate a D-Ala residue into the dermorphin sequence marks a significant advancement in the field of neuropeptides [2].

In the initial clinical assessment, dermorphin demonstrated superiority over morphine in many respects. Unfortunately, from a clinical perspective, the pharmacological data reveal some toxic reactions that restrict the conduct of full-scale clinical trials [3].

Dermorphins continue to hold potential due to their unique structure and functions, paving the way for new modifications for possible practical use as drugs. Therefore, new dermorphin analogs active through noninvasive routes and free from side effects are of great theoretical and practical interest as a foundation for creating a new generation of peptide analgesics and other neurotropic agents with more selective effects [4,5,6,7].

Our literature analysis reveals a trend in dermorphin research, shifting from basic analgesic studies to more clinically oriented research. Early studies focused primarily on the analgesic properties of dermorphin in animal models [2,3,4,5,6,7]. Later studies and reviews have highlighted the potential clinical applications of dermorphin and its analogs, particularly in the management of neuropathic pain and postoperative pain [6,8,9]. Peptides’ low permeability through cell membranes and low oral bioavailability make subcutaneous, intramuscular, or intravenous injections the preferred routes of administration. However, these methods have inherent limitations and the potential for unwanted side effects, highlighting the urgency and importance of developing new, more effective delivery methods [10]. Incorporating cyclic structures is a modern strategy to enhance the enzymatic stability of peptides and thereby make them resistant to proteolytic degradation.

The primary goal of our research was to prepare and select new dermorphin analogs for noninvasive administration. We explored our original method for converting linear peptides into 2,5-diketopiperazine (DKP) derivatives, stabilizing peptide structures by attaching dipeptide DKP elements to the N- or C-termini active peptides or by incorporating an active peptide fragment into a complex structure as a new class of protease-resistant peptidomimetics. We also prepared a library of immuno- and hemoregulatory peptidomimetics [11,12,13,14].

This study describes how our method was applied to preparing neuropeptides from the dermorphin family. The aim of our research was to discover new approaches to synthesizing original dermorphin analogues that exhibit statistically significant activity through non-invasive administration methods. For the first time, the obtained preparations demonstrated this activity following both intranasal and intragastric administration in animals. We publish these original data as a proof of concept for creating an effective analgesic based on our technology [11].

## 2. Results

### 2.1. Synthetic Analogs of Dermorphin and Their Biological Activity In Vitro

Dermorphin (H-Tyr-D-Ala-Phe-Gly-Tyr-Pro-Ser-NH_2_) exhibits a high affinity for μ-opioid receptors and a low affinity for the δ-opioid G-Protein-Coupled Receptors (GPCR) superfamily. The presence of aromatic rings in two amino acid residues—Tyr1 and Phe3—along with the unique amino acid D-Ala_2_, the cyclic amino acid Pro_6,_ and C-terminal Ser-amidation significantly reduces the likelihood of peptide degradation by endopeptidases. Additionally, including the D-Ala residue in the second position highlights its uniqueness.

In this structural and functional study of dermorphin, we synthesized dermorphin (D1), several new linear and cyclic dermorphin analogs (D2–D9), and the N-terminal peptide D10 as reference molecules for comparison (Table 1).

### 2.2. Opioid Activity Studies of New Dermorphin Analogs on Isolated Guinea Pig Ileum

Dermorphin analogs have been tested using the standard guinea pig ileum (GPI) model, The GPI is rich in µ-opioid receptors [15]. Ten peptides were suppressed via electrical stimulation contractions of segment GPI in the studied concentration range (10^−9^ to 10^−6^ M) (Table 1).

The opioid receptor antagonist naloxone (10^−6^ M) prevented or weakened the action of the compounds under investigation, confirming the opioid mechanism of their action. Dermorphin (D1) was used as a reference; peptide D2 showed the highest activity, and cyclopeptides D3 and D4 showed high activity. Thus, new dermorphin analogs can be agonists of µ-type opioid receptors. However, this needs to be verified using molecular modeling methods in further research. Attila M. et al. (1993) investigated the potential binding of 16 dermorphin analogs to µ and δ opioid receptors in brain cells [16]. Using molecular modeling methods, it was found that there is a high probability of such binding for 2 of the 16 studied analogs with µ and δ opioid receptors [16].

The selection of dermorphin analogs is based on the intention to develop peptides that can be administered using noninvasive methods. Peptide D2 contains a D-Ala residue in position 4 instead of a glycine residue, and more hydrophobic methylamine replaces the C-terminal amide of Ser. Peptide D5 was synthesized to compare its activity with that of the amidated peptide D1 and the methyl amidated peptide D2.

According to the literature, introducing an Arg residue in position 2 of the dermorphin (D1) molecule increased the analgesic activity of the N-terminal tetrapeptide D10 [7]. For this purpose, analogs D8 and D9 were prepared to determine their analgesic activity (Table 1).

We used peptide D2 and tetrapeptide D10 as pharmacophores for the synthesis of cyclic peptide mimetics D3, D4, D6, and D7, following the method we developed for synthesizing peptidomimetics based on branched 2,5-DKP.

Thus, the initial screening of the biological activity of dermorphin analogs on the GPI model identified the most active compounds, D2, D3, and D4, which were used to further evaluate their biological activities in comparison with peptide D1 (Table 1).

Experiments using the radioligand method have shown that the affinity of Dermorphins and some of their analogs for µ-opioid receptors is twenty times greater than that of morphine and two times greater than that of the highly selective µ-ligand DAMGO ([D-Ala_2_, N-MePhe_4_, Gly-Ol]—enkephalin) [2].

**Figure 1:**
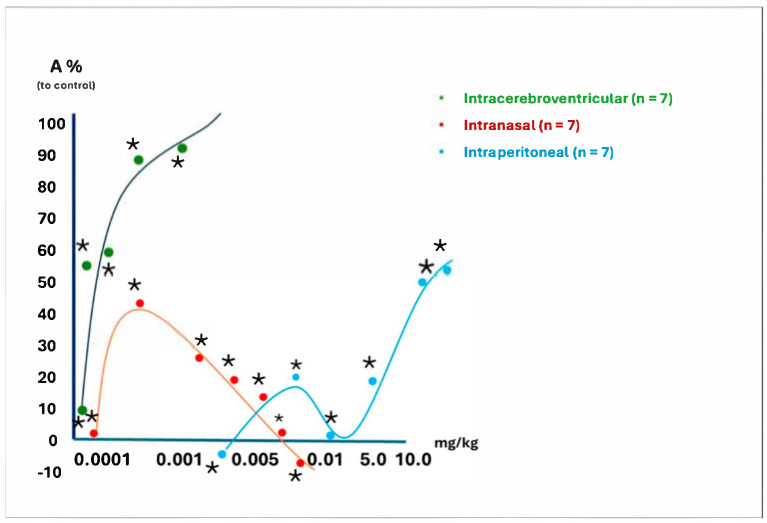
The dose dependence of peptide D2′s analgesic effect in the “water tail-flick” test with different methods of administration (A—analgesic effect, %). *—*p* < 0.05 compared to control; the doses of the substances are expressed in mg/kg of the animal’s body weight on a logarithmic scale (as a percentage of the analgesic effect compared to control animals).

In the experiments, we studied the dose-dependent analgesic activity of the leading peptide D2 using three different administration routes in rats: intracerebroventricular (green curve), intranasal (red curve), and intraperitoneal (blue curve) administration (Figure 1).

Intracerebroventricular administration into the brain’s third ventricle was performed using chronically implanted steel cannulas. When peptide D2 was injected intracerebroventricularly at a dose of 2.5 μg/kg, it resulted in 100% analgesia. This dose induced complete immobility and catatonic effects in the animals. A 1.0 μg/kg dose produced potent analgesia, severe muscle tension, and numbness.

The experiment demonstrated that the dose-dependent analgesic effect of peptide D2, administered intranasally, is complex and multifaceted. A smaller dose of 0.5 µg/kg resulted in 40% analgesia and a short-term state of stupor. The maximum long-lasting analgesia was observed at a dose of 1.0 µg/kg, which induced complete stupor.

The analgesic effect of peptide D2 after intraperitoneal administration at a 5.0 mg/kg dose was over 50%.

### 2.3. Time-Dependent Analgesic Activity Study of Peptides D1 and D2

**Figure 2:**
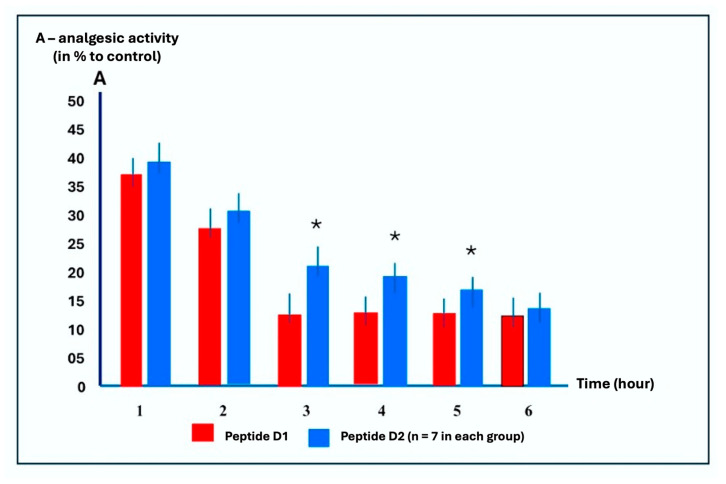
Time dependence of peptide D1 and D2 analgesic effects on the intraperitoneal administration in the “water tail-flick test. *—*p* < 0.05 compared with D2 to D1.

The unique structure and functions of dermorphin open the possibility of new modifications for possible practical use as a drug, including the duration of analgesia. At the next stage of the work, we compared the duration of the analgesic effect of the most active dermorphin analog, peptide D2, with that of dermorphin (D1) (Figure 2).

Figure 2 shows the results of a comparative study on the time-dependent analgesic activity of peptides D1 and D2 following intraperitoneal injections. The experimental design included a control group, relative to which the action of peptides is indicated in Figure 2. The control involved the administration of saline solution to animals. The peptides were diluted with saline solution and injected into the animals according to the same scheme as the saline solution. In this case, the control group acted as a placebo analog.

The intraperitoneal administration of peptide D1 at an optimal dose of 5 mg/kg results in maximal analgesia, which lasts approximately 1 h. In contrast, the analgesic effect of D2 at a 5 mg/kg dose lasts significantly for up to 5 h before pain stimulation (Figure 2).

Thus, these tests demonstrated that peptide D2 is a long-acting, potent analgesic agent that is active upon intranasal administration.

### 2.4. Study Animals’ Behavioral Responses to Peptide D2 in Rats Using the “Open Field” Test with Intranasal Administration

A noticeable suppression of almost all recorded indices characterizing locomotor activity and some unconditioned responses (defecation and urination) was observed in the intact (untainted) group of animals that received intranasal injections of vehicle (0.05 ml of physiological solution).

Such reactions in the control (trained) animals resulted in less pronounced stress following the painful irritation associated with administration. In animals receiving peptide D2 at doses of 15, 50, and 150 μg/kg, significant additional suppression of locomotor activity, but not urination, was observed compared with the control group. In group (1), there was a noticeable suppression of almost all recorded indices. In group (2), the recorded suppression indicator’s reaction was less pronounced.

When peptide D2 was introduced at doses of 15, 50, and 150 μg/kg to animals in groups (3), (4), and (5), significant suppression was observed compared with the control group. Higher doses of the drug revealed a greater depressant effect, with statistically significant dose-dependent suppression observed in groups (3), (4), and (5) (Table 2).

### 2.5. Analgesic Action of Peptides D1, D2, D3, and D4 in Mice

After studying the activity of peptide D2, we conducted a comparative analysis of the activity of peptides D2, D3, and D4. The analgesic activity of these peptides was evaluated with intraperitoneal and intragastric administration using the “tail-flick” and ”hot plate” tests [17].

Dermorphin shows a higher selectivity for µ-opioid receptors than morphine; it is a full agonist for µ-opioid receptors, whereas morphine is a partial agonist for that receptor. In the opioid receptor binding assay using the mouse brain, dermorphin shows a very high affinity for µ-opioid receptors with a low affinity for δ-opioid and κ receptors [18].

The synthesized analogs D2, D3, and D4 were compared with dermorphin (peptide D1). A well-known opioid analgesic, tramadol, at a dose of 50 mg/kg, was used as an analgesic of another chemical group, because in the “hot-plate” test, similar to this dose, tramadol produces a significant analgesic effect in male BALB/c [19].

It is common knowledge that the primary mechanism of analgesic activity of tramadol is the agonist effect on opioid mu, delta, and kappa receptors, as well as the high mu-opioid activity of the primary metabolite of tramadol (o-desmethyltramadol) in the central nervous system. Inhibition of norepinephrine and serotonin reuptake in the descending pathways of the endogenous antinociceptive system is of secondary importance. In addition, tramadol turned out to be more accessible for use in experimental purposes compared to, for example, morphine or fentanyl, which have only the properties of opioid agonists [18].

**Figure 3:**
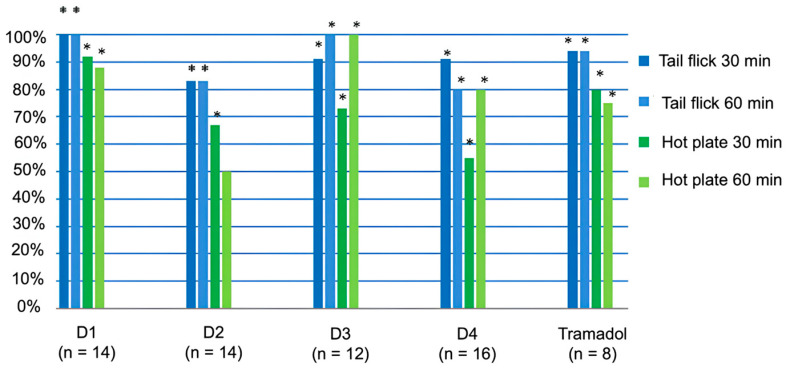
Results of the assessment of the analgesic action of peptides following intraperitoneal injection. *—*p* ≤ 0.05; the exact Fisher criterion was applied for comparison with the control group (which received the solvent). The vertical axis displays the percentage of mice showing a 50% or greater significant analgesic effect compared with the control group. All substances were injected at a dose of 50 mg/kg.

The threshold value of the analgesic action of peptides D1-D4 was determined using the ‘tail flick’ and ‘hot plate’ methods, as shown in Figure 3. According to the definition of the Washington State Opioid Dosing Guideline, the threshold value of the analgesic effect of a substance is defined as the dose required to manifest its 50% analgesic activity [20,21]. The red horizontal line in Figure 3 demonstrates the threshold value of the analgesic action. Values above this line reflect exceeding the threshold value of the analgesic effect of peptides.

As shown in Figure 3, peptides D3 and D4 exhibited high analgesic activity upon intraperitoneal administration, comparable with that of D1, D2, and tramadol in the “tail flick” test. All substances at a dose of 50 mg/kg had a pronounced analgesic effect in both tests (the dose of 50 mg/kg is the average for mice).

In the “hot plate” test, D3 demonstrated the highest activity for 60 min, practically comparable to tramadol; D2 showed significant activity only at 30 min, while D4 was moderately active.

**Figure 4:**
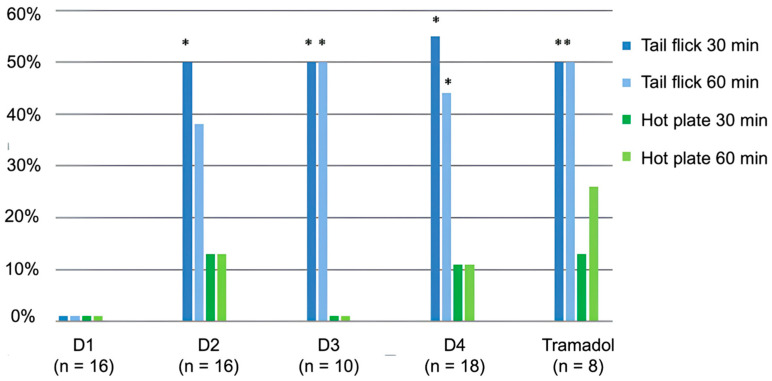
The analgesic effect of synthesized peptides after intragastric administration in mice. *—*p* ≤ 0.05, the exact Fisher criterion was used for comparison with the control group (which received the solvent). The vertical axis shows the percentage of mice exhibiting a 50% or more significant analgesic effect compared with the control group. All substances were administered at a dose of 50 mg/kg body weight.

The analgesic properties of peptides administered via the gastrointestinal route are shown in Figure 4.

During intragastric administration, D1 did not exhibit analgesic action. In contrast, D2 was active in the “tail-flick” test only 30 min after administration, while peptides D3 and D4 were active after 30 and 60 min, respectively, similar to tramadol (Figure 4). There was no significant analgesic effect of peptides D2, D3, D4, or tramadol in the “hot-plate” test.

The analgesic peptides D3 and D4 demonstrated statistically significant activity following non-invasive intragastric administration.

## 3. Discussion

Over the decades, various synthetic strategies have been developed for the preparation of modified neuropeptides, resulting in numerous analogs of peptidomimetics. Systemic administrations of a few opioid peptidomimetics have proved that peripheral administration provides safe analgesia with minimal side effects in the CNS. Further efforts, however, in transforming the molecules to opioid peptide-based drugs with improved safety profiles are still critical, and new formulations or routes of administration may quicken the process.

Due to their difficulty in crossing cell membranes, neuropeptides primarily interact with *extracellular* targets, although interaction with intracellular targets remains challenging for peptide drugs. Similar challenges exist for peptide interactions with *intracellular* central nervous system (CNS) targets. The selectivity advantage of peptides is more pronounced when considering the density and subtype diversity of receptors in the brain, which are modulated by neuropeptides [22].

The implementation of new administration routes for a broad range of peptides, such as cell-penetrating and stapled peptides, is currently in progress. At the same time, the intranasal delivery route is being explored, and oral administration is expected to emerge soon [23].

Cyclization has been a significant tool for developing conformationally constrained structures for opioid receptors. Limiting the number of conformations enhances the ability to correctly position the backbone and/or side chains for the receptors, improving receptor selectivity and specificity, bioavailability, and metabolic stability.

The basic analgesic properties of dermorphin remain unique, despite the considerable time that has passed since initial studies on this neuropeptide. Over the decades, numerous analogs, conjugates with various carriers, and some cyclic analogs with MOR activity have been synthesized. The aim of our research was to develop new synthetic approaches for the preparation of novel dermorphin analogs that can be administered non-invasively. We obtained these original data as proof of concept for creating an effective analgesic based on our technology [11]. The a proof-of-concept strategy involves a step-by-step study of promising molecules, starting with experiments to optimize synthesis, and verification of primary data on biological activity in vitro and in vivo. The main difference in our studies is that we employed a method for obtaining cyclic compounds with different biological activities that do not contain non-peptide scaffolds or other fragments [11]. These experimental synthetic studies of dermorphins are divided into two parts: the rational design of conformationally restricted dermorphin analogs that are stable at non-invasive administration, and confirmation of non-invasive activity in commonly used tests. As a result of this step, we prepared, for the first time, a peptidomimetic analog of dermorphin, active in intragastric administration. We conducted a comparative analysis of published dermorphin analogs, specifically examining the route of administration, analgesic activity, and structure-related advantages, and we did not find a description of cyclic analogs of dermorphin that are active when administered orally (intragastric).

In our research, we used C-terminal hydrophobic modifications of linear peptides. Another alternative is to convert linear peptides into 2,5-DKP derivatives, resulting in protease-resistant peptidomimetics [24]. We expanded our structure-activity relationship (SAR) research on 2,5-DKP-related branched peptidomimetics to study neuropeptides as analgesics and behavioral regulators. The neurotropic activity of 2,5-DKP compounds is documented in the literature [25,26]. We also investigated opioid and analgesic activities of dermophyllines modifications.

The classical solution method was used to synthesize new linear and cyclic analogs of dermorphin. The leading compounds D2, D3, and D4 (Table 1) were selected for further SAR studies after determination of their in vitro analgesic activity. Peptide D1 (dermorphin) was used as the standard of comparison.

The leading peptide, D2, was designed for potential noninvasive applications, such as intranasal or oral administration. Inserting a D-Ala residue instead of Gly at position four and substituting Ser-NH_2_ with Ser-NH-Me increased the hydrolytic stability of peptide D2.

Peptides D5 and D10 contained D-Ala at position four as part of the development of corresponding analogs. Considering the increasing stability of peptides achieved by synthesizing cyclic neuropeptide analogs, we employed a parametric optimization calculation method to use the main 2,5-DKP cyclopeptide models as scaffolds for attaching peptide D2.

Functional groups (pharmacophores) of branched cyclopeptides readily undergo metabolic transformations; for example, they are hydrolyzed in the organism when attached to the centroid by ester bonds. The modeling of peptide conformations allowed us to isolate the ground states from the conformational ensembles. The starting compounds were peptide structures with a narrow spectrum of conformational energy [8,24].

**Figure 5:**
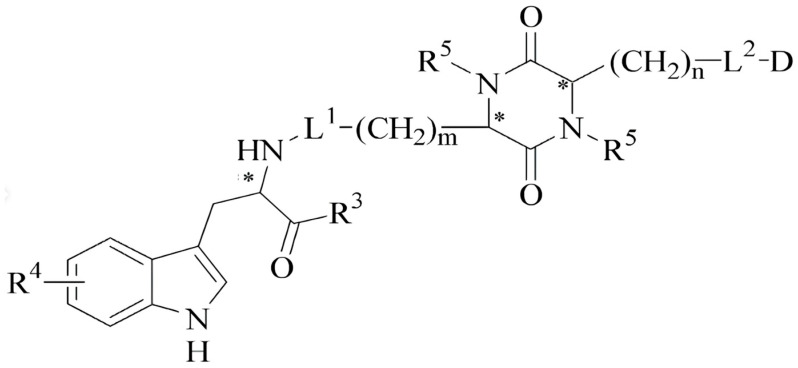
Cyclo-DKP scaffold for the preparation of cyclopeptide conjugates. Cyclodipeptides (2,5-DKP), composed of trifunctional amino acids, contain various functional groups that can be used both for “filling” the target positions (with which this molecule interacts) and as linkers for attaching different pharmacophores [11]. L—linkers, D—derivates of peptides and small molecules, m, n—number of CH_2_-groups, R _1-5_—various substitutes, *—optically active centers.

**Figure 6:**
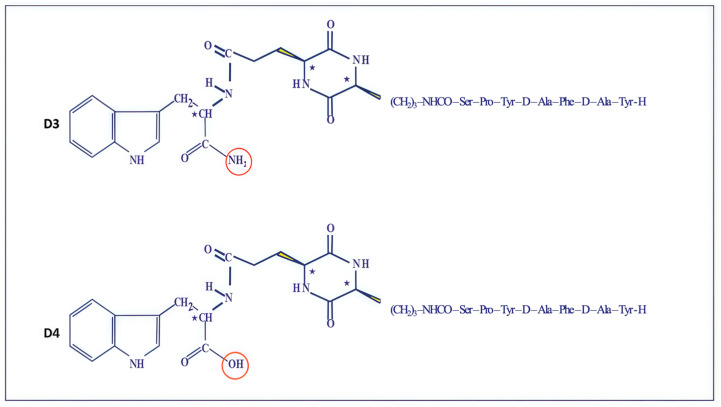
Schematic structures of cyclo-peptides D3 and D4. Cyclo{Lysil-gamma-Glutamyl-Tryptophan-amide)} D3. Cyclo{Lysil-gamma-Glutamyl-Tryptophan-OH)} D4. *—optically active centers.

Initially, we tested this hypothesis by preparing conjugates of the cyclo 2,5-DKP cyclopeptide scaffold (Figure 5). In these chimeric compositions, 2,5-DKP served as a “protecting pharmacophore attachment” to improve the molecule’s hydrolytic stability during oral administration for the modified analogs D3 and D4 [27] (Figure 6).

After oral administration in mice, the LC-MS analysis of these compounds showed that both tested chimeric molecules remained unhydrolyzed [28].

Emerging research in these areas, utilizing novel machine learning and computational modeling techniques, will further enhance the therapeutic potential of neuropeptides in neurological and peripheral diseases [29].

In earlier studies, experiments were conducted to study the activity of dermorphin via various routes of administration in animals. The analgesic effect of dermorphin was tested through intracerebroventricular (ICV) administration in rats using the “hot-plate” and “tail-flick” tests, as well as via the intravenous (IV) route in mice.

Overall, dermorphin produces long-lasting antinociceptive activity [30].

Our experiments demonstrated that the intraperitoneal administration of peptide D2 at a dose of 5 mg/kg resulted in a significantly prolonged decrease in pain sensitivity, lasting much longer than that of D1 (Figure 1).

In another experiment, we evaluated the analgesic activity of peptide D2 in rats using three different routes of administration: intracerebroventricular, intraperitoneal, and intranasal. Dose-dependent intraperitoneal injections caused significant analgesia that lasted five hours after administration with various doses (Figure 2).

The test results showed that the D2 peptide induces an antinociceptive response by acting at the spinal level.

The influence of experimental drugs on animal behavior is an essential step in the preclinical process. Our study employed behavioral tests to assess general locomotor activity levels, anxiety, and willingness to participate in the study. The D2 peptide, administered intranasally at doses of 15, 50, and 150 μg/kg, caused a significant dose-dependent suppression of locomotor activity compared to the control group. At the same time, higher doses of the drug revealed a depressant effect. Peptide D2 was observed to be the most significantly suppressed as almost all recorded indices were noticeably suppressed in the intact group (Table 2).

Suppression of nearly all recorded parameters characterizing locomotor activity (distance traveled), orientational-exploratory behavior (number of dives and stands), and some unconditioned reactions (defecation and urination) was observed. This reaction in the intact animals was due to stress caused by the pain stimulus associated with the peptide administration. The response to the recorded suppression indicator was less pronounced in the control group.

Thus, the presented data indicate that peptide D2, when administered intranasally, suppresses the locomotor and exploratory-orientational activity of rats in the “open field” test.

This behavior study has led to the following conclusions: the drug’s direct effect upon intranasal administration of escalated doses causes a dose-dependent depression of motor activity.

High doses of opioid peptides, such as the D2 peptide, cause central suppression of motor activity, which can manifest as catatonia, stupor, and motor rigidity. These effects complicate the conduct of nociceptive tests, such as the tail-flick test, because animals experiencing motor disturbances may not exhibit the expected response to a painful stimulus. Thus, despite actual pain perception, behavioral indicators may appear as analgesia, leading to false positive conclusions. The scientific literature reports cases where morphine and other opioids simultaneously induced an analgesic effect in the tail flick test [31] and catatonic symptoms, confirming the possibility of confounding analgesic effects with motor inhibition at high doses. Additionally, it is noted that opioid centralization often accompanies motor function suppression, which must be considered when interpreting pain test results. Studies show that suppression of motor activity limits the animal’s ability to demonstrate a pain response, even if nociceptive perception is intact, necessitating the use of additional methods to determine true analgesia [32,33]. Therefore, caution is essential when interpreting nociceptive test results at high doses of peptides or opioids. To avoid false conclusions, the influence of motor inhibition should be accounted for, and functional tests should be supplemented with additional measures capable of distinguishing true analgesia from nonspecific motor dysfunction.

The analgesic activity of peptides D1, D2, D3, and D4 in mice was evaluated using the “tail-flick” and “hot-plate” tests at intraperitoneal and intragastric administration.

Figure 3 (Section 2) presents the results of evaluation results for the analgesic action of peptides compared with tramadol at intraperitoneal injection in the “tail-flick” and “hot-plate” tests. All peptides were active in both tests; at the same time, in the “Tail-Flick” test, the activity of D1 and D3 lasted for 60 min, comparable with tramadol, and in the “Hot Plate” test, D1, D3, and D4 were active as tramadol, and D2 was active only for 30 min.

Figure 4 (Section 2) presents the experimental results of the tested peptides at intragastric applications. At these tests, D1 was inactive; D2, D3, D4, and tramadol were active only in the “Tail-Flick” from 30 (D2) to 60 min (D3, D4, and tramadol); and none of the tested molecules were active on the “hot-plate” test.

The study results of the analgesic activity of cyclic compounds showed hydrolytic stability upon administration. Active peptidomimetics D3 and D4 are administered using our original method of combining linear peptide D2 with 2,5-DKP scaffold, preserving the activity of linear peptide D2.

The precise changes in scaffolds 1 (-NH_2_) and 2 (-OH) structures resulted in different levels of biological effects in both tests.

These experiments confirmed the high, long-lasting analgesic activity of peptide D2 following parenteral, intranasal, and short-term intragastric administration (Figure 6).

The predominant trend in peptide pharmaceutical development is the conversion of linear peptides into various peptidomimetics [27].

To further contextualize our findings, we conducted a comparative analysis of published dermorphin analogs, focusing on structural modifications, administration routes, and analgesic efficacy. Although several cyclopeptides have been synthesized with promising μ-opioid activity, to date, no literature reports have confirmed analgesic activity of cyclic neuropeptide analogs via intragastric routes [30,31,32]. However, the cyclization of small opioid peptides is not straightforward due to their inability to fold into a compact structure (Table 3) [34,35,36].

The current work represents the first generation of cyclic dermorphin analogs (D3 and D4), demonstrating that these 2,5-DKP-cyclized compounds exhibit improved enzymatic stability and retain potent μ-agonist activity in vivo. The design strategy, based on DKP topologies, combines pharmacophore stabilization with resistance to proteolysis, offering a novel approach for the non-invasive administration of peptide pharmaceuticals.

## 4. Materials and Methods

The procedures performed in this study followed the Guide for the Care and Use of Laboratory Animals published by the National Institutes of Health and the “Regulations for Studies with Experimental Animals”, USA (Decree of the Russian Ministry of Health from 12 August 1997, No. 755). The Institutional Ethics Committee of the Shemyakin-Ovchinnikov Institute of Bioorganic Chemistry approved the protocol (Protocol No. 186/2023, dated 8 October 2023).

### 4.1. Animals

The test animals were C57BL/6 and Balb/C female mice, weighing 22–26 g, and Wistar rats, weighing 240–260 g, from the “Stolbovaya” Animal Breeding Center. All animals were quarantined for two weeks before the experiments. Only animals that appeared healthy were used for the study. A basal diet and water were available ad libitum. Environmental management included temperature regulation, ventilation, humidity control, lighting, bedding, and environmental enrichment. Animals were accommodated with an automated 12-h light/12-h dark cycle. Heating and cooling were electronically controlled to maintain the animal room temperature between 18 and 22 °C.

### 4.2. Synthesis of Linear Peptides

The peptides were synthesized in solutions using the classical method with maximum protection of the functional groups and purified using preparative high-performance liquid chromatography (HPLC). The purity and structure of the final products were controlled using analytical HPLC-MS spectroscopy.

### 4.3. Synthesis of Cyclopeptides and Development of Noninvasive Forms of Dermorphin Analogs

**Figure A1:**
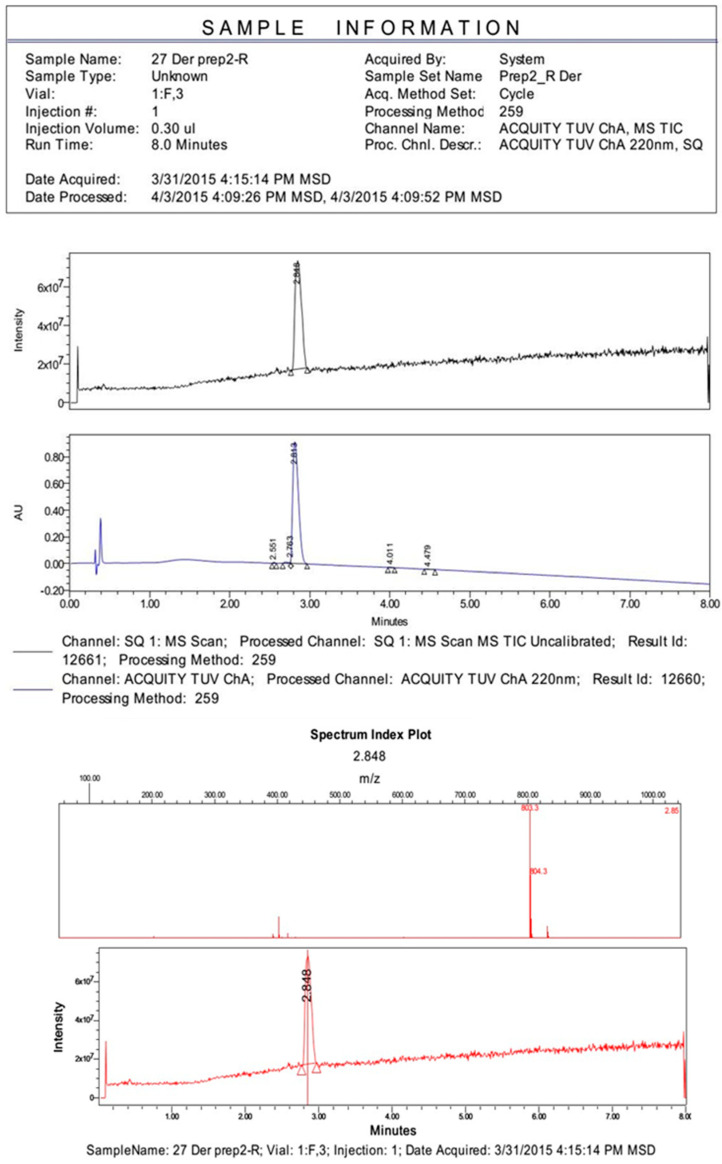
Analytical UPLC-MS for peptide D1.

The method for converting linear dipeptides into (2,5-diketopiperazine (2,5-DKP) derivatives enables the stabilization of peptide structures and the incorporation of an active cyclic fragment into a complex structure (Figure A1, Table A1). This approach represents a new group of protease-resistant peptidomimetics [11].

**Figure 7:**
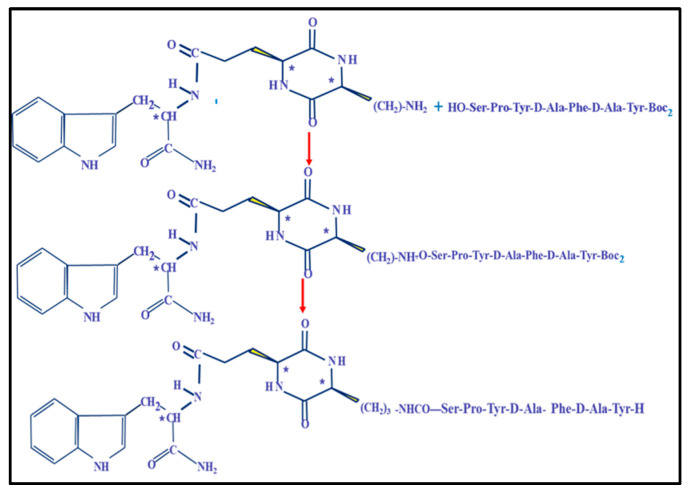
The synthetic route of cyclopeptide D3. A detailed description of the peptides and peptidomimetics synthesis is presented in Appendix A. *—optically active centers.

**Figure A3:**
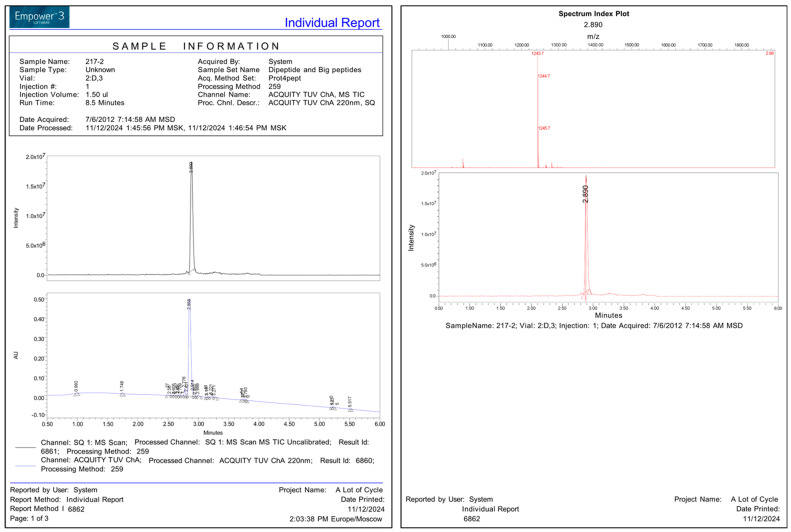
Analytical UPLC-MS for peptide D3.

The protected derivative of peptide D5 (Tyr-D-Ala-Phe-D-Ala-Tyr-Pro-Ser-OH, A1) was used as an intermediate of peptide D2 for the synthesis of peptidomimetics D3 and D4. It was combined with two 2,5-DKP derivatives: Cyclo[Lys-Glu(Trp-OH)] and Cyclo[Lys-Glu(Trp-NH_2_)] (Figure 7 and Figure A3) [12,13].

### 4.4. Peripheral Opioid Activity of Peptides on Isolated Guinea Pig Ileum

The peripheral opioid activity of the peptides was assessed according to known methodology [15]. A segment of guinea pig ileum (approximately 1 cm long) was placed into a 10 mL organ bath containing Krebs solution at 34 °C. Single pulses stimulated the segment of the ileum (1 g) with a duration of 1 ms and a frequency of 0.1 Hz at 80 V. Contractions were recorded in isometric mode using a K30 sensor (Hugo Sachs Elektronic KG, March, Germany).

All tests for each molecule were performed in duplicate. Based on the data obtained, dose-effect curves were plotted, and the IC_50_ index was determined as the concentration of the substance that causes 50% of the maximal inhibitory effect.

### 4.5. Analgesic Action of Peptide D2 Using Different Routes of Administration on Rats

#### 4.5.1. Comparative Study of Time-Dependent Analgesic Activity of Peptides D1 and D2 at Intraperitoneal Injection

The strength and duration of the analgesic action of peptides D2 and D1 were determined using the “water immersion tail flick” test, administered at a dose of 5 mg/kg. [17].

The animal was placed in a plastic chamber, and the tail was immersed 5 cm in water at 56 ± 1 °C. Analgesic activity was assessed every 10 min for each hour after administration to determine the duration of the drug’s action. The test was duplicated for five animals in each case. The control group received tramadol at a dose of 50 mg/kg. [19].

The analgesic effect (A) was calculated as the average of the A values corresponding to the 20th to 60th minute after the peptides were administered, according to the following Equation (1):

A = (t_1_ – t_0_)/(t_max_ − t_1_) × 100%, (1)

where

t_1_—time of relief from pain stimulus after the introduction of substances,
t_0_—time of relief from pain stimulus as the baseline,
t_max_—maximal duration of pain stimulus (30 sec).

#### 4.5.2. The Dose-Dependent Activity of D2 at Three Administration Routes in Rats

Administration was carried out intracerebroventricularly (*n* = 7), intranasally (*n* = 7), and intraperitoneally (*n* = 7). Intracerebral administration (10 μL in saline per animal) was performed into the brain’s third ventricle was performed through chronically implanted steel cannulas. The initial pain sensitivity was determined for one hour in each group.

Intranasal administration was carried out using a soft plastic catheter attached to a microsyringe. A dose of 0.5 µg/kg caused 50% analgesia and a short-term state of stupor. The maximum long-lasting analgesic effect was observed at a dose of 1.0 µg/kg. The analgesic effect of peptide D2 after intraperitoneal administration of 5.0 mg/kg was 40% less than the 100% effect seen in the control group.

All tests for each molecule were performed in duplicate. The data obtained were processed and analyzed using nonparametric statistical methods.

### 4.6. The Effect of D2 on the Central Nervous System

#### 4.6.1. The Effect of Intranasal Administration of D2 on the Behavioral Responses of Rats in the “Open Field” Model

The experiments were conducted in an automated “open-field” chamber that registered and processed computer data. They were conducted in two replicates on 64 male Wistar rats weighing 240–260 g. The “open-field” is a round arena with a diameter of 80 cm, lined with three concentric circles and eight diameters. During the experiment, the rat was placed in the center of the arena. Motor activity (measured as the number of segments passed), the number of departures from the arena wall, vertical activity (the number of rises on the hind legs), and the number of touches of the muzzle with the paws (washing) were visually assessed for two minutes. A 500-watt household electric bell and a 15-watt red lamp were placed 80 cm above the arena. The study was conducted in silence and under the light of the red lamp.

Animals were divided into groups: untrained intact animals and trained experimental animals. All animals were divided into five groups:

1. In the intact group, animals without training were injected with 0.05 mL of physiological solution one minute before being placed in the “open field.” (n = 33).
2. The control group consisted of animals adapted after a 5-day administration of 0.5 mL of saline (n = 10).
3. Single drug action at a 15 μg/kg dose was administered to adapted animals (n = 7).
4. Adapted animals (n = 7) received a 50 μg/kg dose of a single drug action.
5. Single drug action at a 150 μg/kg dose was administered to adapted animals (*n* = 7).

#### 4.6.2. The Analgesic Action of Peptides D1, D2, D3, and D4 in Mice After Intraperitoneal and Intragastric Administration

Two tests, “tail-flick” and “hot plate,” were used to evaluate the analgesic action of peptides administered intraperitoneally and intra-gastrically [17].

The pain stimulus was applied locally to the tail as radiant heat using a Tail Flick Analgesia Meter (Hugo Sachs Elektronik, Germany). A tail-flick latent period of 6 s was considered the maximum time for applying the stimulus (Equation (1): t_max_—6 s, maximal duration of pain stimulus).

The control groups consisted of mice injected with solvent. The tested substances were administered to the animals intraperitoneally and intragastrically in a volume of 0.1 mL of saline per 10 g of body weight. The pain response threshold was measured before and 30 and 60 min after administration.

The “hot plate” test evaluates the pain reaction to thermal irritation of the mouse paw. The Hot Plate Model 7280 (Ugo Basile S.R.L., Gemonio, Italy) analgesimeter was used for this test. The maximal duration of the pain stimulus (thermal irritation) was set at 60 s.

In both tests, the final indicator of analgesic action was defined as a 50% or more significant effect on the number of animals in each experimental group, expressed as a percentage of the total number of animals in that group.

Experimental groups for intraperitoneal administration included:

Control (saline)—*n* = 11, analgesic action was not observed;
D1 (50 μg/kg)—*n* = 16;
D2 (50 μg/kg)—*n* = 16;
D3 (50 μg/kg)—*n* = 10;
D4 (50 μg/kg)—*n* = 18;
Tramadol (50 μg/kg)—*n* = 18.

Experimental groups for intragastrical administration included:

Control (saline)—*n* = 9, analgesic action was not observed;
D1 (50 μg/kg)—*n* = 14;
D2 (50 μg/kg)—*n* = 14;
D3 (50 μg/kg)—*n* = 12;
D4 (50 μg/kg)—*n* = 16;
Tramadol (50 μg/kg)—*n* = 18.

The tramadol dose was chosen based on the published data [37]

**Figure 8:**
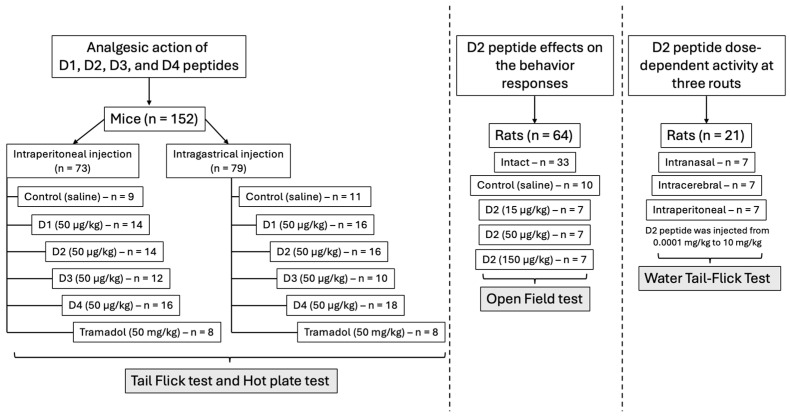
Schematic design of the experiment.

The experimental design scheme is presented in Figure 8.

### 4.7. Statistical Analysis

The data obtained were analyzed using Student’s *t*-test for behavioral tests, the exact Fisher test for analgesic activity, and the statistical software STATGRAF 1.0.6 for peptidomimetic calculations.

## 5. Conclusions

Peptides show great promise in targeting extra- and intracellular delivery of next-generation biotherapeutics. However, their susceptibility to proteolysis in biological systems is a critical limitation. Numerous chemical strategies have been developed to enhance resistance to proteolysis and overcome this challenge.

Structure-activity relationship (SAR) studies have demonstrated the high activity of new analogs of dermorphin, specifically peptide D2 and peptidomimetic D3, at parenteral, intranasal, and intragastric administration routes.

The toxicological and safety studies were performed as part of preclinical studies based on the encouraging experimental results regarding the high hydrolytic stability of peptide D2 at intranasal and intragastric administration.

We plan to conduct further preclinical and clinical studies of our peptidomimetic D3 as analgesic drugs in noninvasive oral solid-finished forms.

## Figures and Tables

**Table 1:** Chemical structures of the synthetic dermorphin analogs and index IC_50_ mol/l (in the right column) characterizing their opioid activity in isolated preparations of guinea pig ileal smooth muscle (details below).

Code	Structure	Index IC_50_ mol/L *
D1	H-Tyr-D-Ala-Phe-Gly-Tyr-Pro-Ser-NH_2_ (dermorphin)	2.5 × 10^−9^
D2	H-Tyr-D-Ala-Phe-D-Ala-Tyr-Pro-Ser-NH-CH_3_	9.5 × 10^−9^
D3	Cyclo-[L-Lys(H-Tyr-D-Ala-Phe-D-Ala-Tyr-Pro-Ser)-L-Glu(L-Trp-NH_2_)]	3.3 × 10^−8^
D4	Cyclo-[L-Lys(H-Tyr-D-Ala-Phe-D-Ala-Tyr-Pro-Ser)-L-Glu(L-Trp-OH)]	1.1 × 10^−8^
D5	H-Tyr-D-Ala-Phe-D-Ala-Tyr-Pro-Ser-OH	7.6 × 10^−7^
D6	Cyclo-[L-Lys(H-Tyr-D-Ala-Phe-D-Ala)-L-Glu(OH)]	5.7 × 10^−7^
D7	Cyclo-[L-Lys(H-Tyr-D-Ala-Phe-D-Ala)-L-Glu-(L-TrpOMe)]	2.0 × 10^−6^
D8	Arg-Tyr-D-Ala-Phe-Gly-OH	3.0 × 10^−6^
D9	H-Arg-Tyr-D-Ala-Phe-D-AlaOH	1.0 × 10^−6^
D10	H-Tyr-D-Ala-Phe-D-Ala-OH	1.0 × 10^−6^

* IC_50_ (half maximal inhibitory concentration) is a quantitative measure of the molar concentration of an inhibitory substance in vitro, which inhibits a given biological component by 50% [14].

**Table 2:** Effect of D2 on the behavioral responses of intact animals in an “open field” test.

Action	Intact (*n* = 33)	Control (Trained *n* = 10)	Peptide D2 (dose, µg/kg)		
			15 (*n* = 7)	50 (*n* = 7)	150 (*n* = 7)
Path	100.75 ± 6.31	65.80 ± 13.00 *	23.83 ± 7.16 **	21.80 ± 6.16 **	14.80 ± 3.80 **
Dive	25.63 ± 2.04	11.70 ± 2.12 *	6.17 ± 1.63 **	3.40 ± 0.76 **	2.90 ± 0.99 **
Stand	33.51 ± 2.42	16.30 ± 4.37 *	8.83 ± 3.03 **	4.50 ± 1.35 **	4.70 ± 1.45 **
Grooming	7.16 ± 0.68	6.80 ± 1.82 *	1.58 ± 0.42 **	0.90 ± 0.46 **	1.00 ± 0.39 **
Defecation	2.73 ± 0.23	2.10 ± 0.43 *	0.92 ± 0.26 **	1.10 ± 0.18 **	0.60 ± 0.22 **
Urination	0.820 ± 0.12	0.30 ± 0.15	0.25 ± 0.13	0.20 ± 0.13	0.10 ± 0.10
Path	100.75 ± 6.31	65.80 ± 13.00 *	23.83 ± 7.16 **	21.80 ± 6.16 **	14.80 ± 3.80 **
Dive	25.63 ± 2.04	11.70 ± 2.12 *	6.17 ± 1.63 **	3.40 ± 0.76 **	2.90 ± 0.99 **
Stand	33.51 ± 2.42	16.30 ± 4.37 *	8.83 ± 3.03 **	4.50 ± 1.35 **	4.70 ± 1.45 **

*—Significance of differences with the intact group (*—*p* <0.05; *); **—*p* <0.01; ** compared with the control group.

**Table 3:** The cyclic structures of Dermorphin analogs.

Code	Structure
D1	H-Tyr-D-Ala-Phe-Gly-Tyr-Pro-Ser-NH_2_ (dermorphin)
D2	H-Tyr-D-Ala-Phe-D-Ala-Tyr-Pro-Ser-NH-CH_3_
D3	Cyclo-[L-Lys(H-Tyr-D-Ala-Phe-D-Ala-Tyr-Pro-Ser)-L-Glu(L-Trp-NH_2_)]
D4	Cyclo-[L-Lys(H-Tyr-D-Ala-Phe-D-Ala-Tyr-Pro-Ser)-L-Glu(L-Trp-OH)]
D6	Cyclo-[L-Lys(H-Tyr-D-Ala-Phe-D-Ala)-L-Glu(OH)]
D7	Cyclo-[L-Lys(H-Tyr-D-Ala-Phe-D-Ala)- L-Glu-(L-TrpOMe)]
	Tyr-c2,5 (-S-) [*D*Val-Gly-he-*D*Ala]-OH
	Tyr-c2,5 (-S-) [DAla-Gly-Phe-DAla]-OH
	Tyr-c2,5 (-CH2CH2-) [DAla-Gly-Phe-Ala]-NH_2_
	Tyr c2,5 (-cisCH=CH-) [DAla-Gly-Phe-DAla]-OH

**Figure A2:**
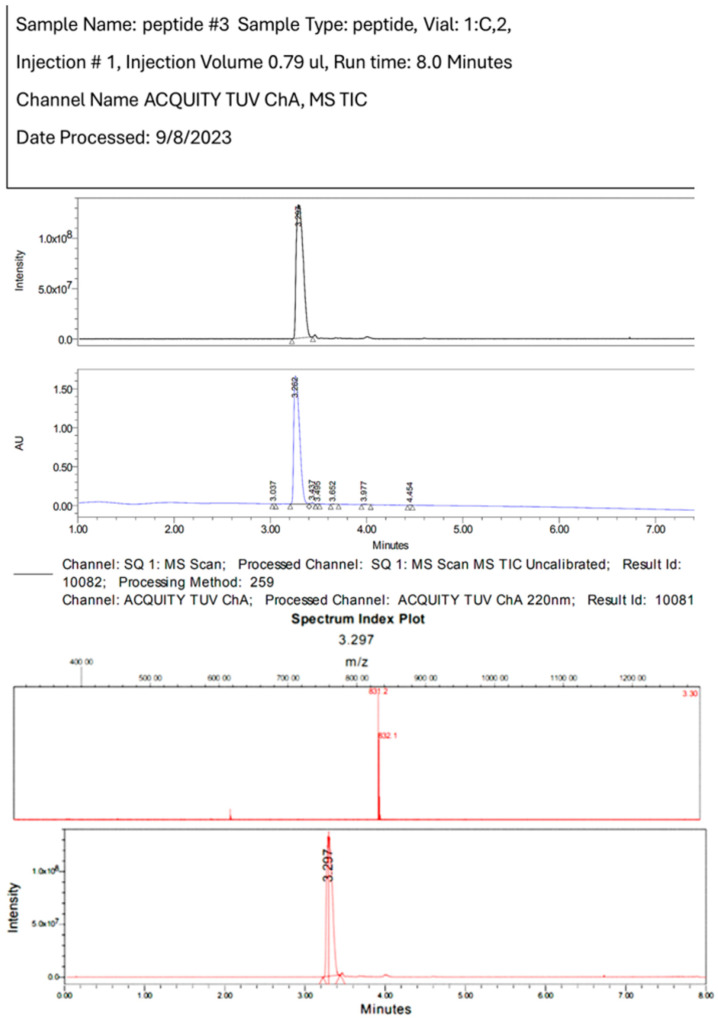
Analytical UPLC-MS for peptide D2.

## Data Availability

Data is unavailable due to privacy or ethical restrictions.

## Appendix A

### Appendix A.1

In this work, we used derivatives of L-amino acids from Fluka (Buchs, Switzerland), Bachem (Bubendorf, Switzerland), Merck (Darmstadt, Germany), and Reanal (Budapest, Hungary), as well as solvents and reagents from Galakhim (Moscow, Russia). and Khimmed (Saint Petersburg, Russia). TLC was carried out on DC-Fertigplatten SIL G-25 plates from Macherey-Nagel (Duren, Germany) using the following solvent systems: chloroform–ethyl acetate–methanol (6:3:1) (A); chloroform–ethyl acetate–methanol–acetic acid (60:30:10:2) (B); chloroform-methanol-acetic acid (32%) (65:45:20) (C). Derivatives of amino acids and peptides were detected with a standard ninhydrin reagent (chromatograms with Boc-protected amino derivatives were pre-exposed to trifluoroacetic acid vapors).

Analytical chromatography was performed on an Alliance chromatograph by Waters (USA) with a Symmetry C18 5 μm column (3.9 × 150 mm). Buffer A consisted of 0.05% TFA, while Buffer B contained 0.025% TFA in 80% CH_3_CN, which were used as eluents. Elution was conducted using a concentration gradient of Buffer B from 0% to 80% in Buffer A over 30 min, with a flow rate of 1.0 mL/min. Detection was performed at three wavelengths: 210, 254, and 280 nm.

Preparative chromatography was performed on a Waters Prep LC System using a 50 × 250 mm column, with Diasorb-130 C16T (6 μm) sorbent from Biohimmak ST, Russia. The eluent consisted of Buffer A (0.1% AcOH and 2% isopropanol in water) and Buffer B (isopropanol), with a gradient from 0% to 70% Buffer B in Buffer A. The flow rate was 50 mL/min, and detection occurred at 280 nm.

Mass spectrometric analyses were performed on a Waters Aquity H-Class instrument using a column measuring 2.1 × 50 mm (Waters Aquity UPLC BEH C18, 1.7 μm), equipped with a Waters Aquity UPLC SQ Detector mass spectrometric detector.

**Table A1:** Chemical characteristics of synthetic peptides.

Code	Structure	MW	HPLCtr	MS
D1	H-Tyr-D-Ala-Phe-Gly-Tyr-Pro-Ser-NH_2_ (dermorphin)	802	3.14	803
D2	H-Tyr-D-Ala-Phe-D-Ala-Tyr-Pro-Ser-NH-CH_3_	830	3.28	831
D3	Cyclo-[L-Lys(H-Tyr-D-Ala-Phe-D-Ala-Tyr-Pro-Ser)-L-Glu- (L-Trp-NH_2_)]	1242	19.0	1243
D4	Cyclo-[L-Lys(H-Tyr-D-Ala-Phe-D-Ala-Tyr-Pro-Ser)-L-Glu-(L-Trp-OH)]	1241	16.0	1242
D5	H-Tyr-D-Ala-Phe-D-Ala-Tyr-Pro-Ser-OH	817	3.19	818
D6	Cyclo-[L-Lys(H-Tyr-D-Ala-Phe-D-Ala)-L-Glu(OH)]	801	17.4	802
D7	Cyclo-[L-Lys(H-Tyr-D-Ala-Phe-D-Ala)-L-Glu- (L-TrpOMe)]	986	19.8	987
D8	Arg-Tyr-D-Ala-Phe-Gly-OH	613	1.76	614
D9	Arg-Tyr-D-Ala-Phe-D-Ala-OH	627	1.79	628
D10	H-Tyr-D-Ala-Phe-D-Ala-OH	471	2.68	472

### Appendix A.2

A typical example of the method for the synthesis of peptides D1 and D2 is given in the following.

1. The synthesis of Boc-Phe-D-Ala-OBzl.

A total of 0.6 g (0.003 mol) of Boc-Phe-OH is dissolved in 10 mL of dioxane, equimolar amounts of TBTU and NMM are added, and the solution is stirred for 3 min. Then, a solution of 1.6 g (0.0031 mol) of Tos. H-D-Ala-OBzl in 15 mL of dioxane are added. The solution is stirred with a magnetic stirrer for 2 h, maintaining a pH of 7–8. The reaction progress is monitored with TLC (system A). After the reaction is complete, the solution is evaporated in a vacuum. Next, 50 mL of EtOAc/H_2_O is added to the residue, and transferred it to a separatory funnel, and the pH is adjust to 3. The organic layer is washed with 5% H_2_SO_4_, followed by H_2_O, and then with 5% NaHCO_3_, followed by H_2_O. The solution is dried over Na_2_SO_4_ and then evaporated in a vacuum. The oily residue is dissolved in 30 mL of ethyl acetate, and then ether is added until opalescence is achieved. The solution with the residue is left at +8 °C overnight in the refrigerator. The precipitate is filtered off, washed with ether and hexane, and dried in air. Rf = 0.8 (A).

Yield: 1.8 g (95%).

Boc-Phe-Gly-OBzl (Rf = 0.8 (A)) is obtained similarly.

2. The synthesis of TFA. H-Phe-D-Ala-OBzl

First, 4.9 g (0.009 mol) of the peptide obtained in the previous step is dissolved in 50 mL of 80% trifluoroacetic acid (TFA) and stirred for 45 min. The reaction progress is monitored with TLC (system A). After the reaction, the solution is evaporated in a vacuum and precipitated with ether. Rf = 0.22 (A). The precipitate is filtered off and air-dried.

Yield: 4.5 g (92%).

TFA. H-Phe-Gly-OBzl (Rf = 0.8 (A)) is obtained similarly.

3. The synthesis of Boc-D-Ala-Phe-D-Ala-OBzl

A total of 0.6 g (0.003 mol) of Boc-D-Ala-OH is dissolved in 10 mL of dioxane, add equimolar amounts of TBTU and NMM are added, and the solution is stirred for 3 min. Then, a solution of 1.6 g (0.0031 mol) of TFA.H-Phe-D-Ala-OBzl in 15 mL of dioxane is added. with the solution is stirred a magnetic stirrer for 2 h, maintaining a pH of 7–8. The reaction progress is monitored using TLC (system A). After the reaction is complete, the solution is evaporated in vacuo. Next, 50 mL of EtOAc/H_2_O is added to the residue and transferred it to a separatory funnel, and the pH is adjusted to 3. The organic layer is washed with 5% H2SO_4_, followed by H_2_O, then 5% NaHCO_3_, and finally H_2_O. The solution is dried over Na_2_SO_4_, then evaporated in vacuo. The only residue is dissolved in 30 mL of ethyl acetate, and ether is added until an opalescent solution is achieved. The solution is left with the precipitate at +8 °C overnight in the refrigerator. The precipitate is filtered off, washed with an ether/hexane mixture, and dried in the air. Rf = 0.8 (A).

Yield: 1.8 g (95%).

Boc–D-Ala-Phe-Gly-OBzl (Rf = 0.8 (A)) were obtained similarly. Boc-D-Ala-Gly-OBzl (Rf = 0.8 (A)).

4. The synthesis of TFA. H-Phe-D-Ala-OBzl

First, 4.9 g (0.009 mol) of the peptide obtained in the previous step is dissolved in 50 mL of 80% trifluoroacetic acid (TFA) and stirred for 45 min. The reaction progress is monitored using TLC (system A). After the reaction, the solution is evaporated in a vacuum and precipitated with ether. Rf = 0.22 (A). The precipitate is filtered off and dried it in the air.

Yield: 4.5 g (92%).

TFA. H-Phe-Gly-OBzl (Rf = 0.8 (A)) is obtained similarly.

5. The synthesis of Boc-D-Ala-Phe-D-Ala-OBzl

A total of 0.6 g (0.003 mol) of Boc-D-Ala-OH is dissolved in 10 mL of dioxane, add equimolar amounts of TBTU and NMM, and the solution is stirred for 3 min. Then, a solution of 1.6 g (0.0031 mol) of TFA.H-Phe-D-Ala-OBzl in 15 mL of dioxane is added. The solution is stirred with a magnetic stirrer for 2 h, maintaining a pH of 7–8. The reaction progress is monitored using TLC (system A). After the reaction is complete, the solution is evaporated in vacuo. Next, 50 mL of EtOAc/H_2_O is added to the residue and transferred it to a separatory funnel, and the pH is adjusted to 3. The organic layer is washed with 5% H_2_SO_4_, followed by H_2_O, then 5% NaHCO_3_, and finally H_2_O. The solution is dried over Na_2_SO_4_, then evaporated in vacuo. The oily residue is dissolved in 30 mL of ethyl acetate, and then ether is added until opalescence is achieved. The solution with the residue is left at +8 °C overnight in the refrigerator. The precipitate is filtered off, washed with ether and hexane, and dried in air. Rf = 0.8 (A).

Yield: 1.8 g (95%).

Boc–D-Ala-Phe-Gly-OBzl (Rf = 0.8 (A)) and Boc-D-Ala-Gly-OBzl (Rf = 0.8 (A) were obtained similarly.

6. The synthesis of TFA. H-D-Ala-Phe-D-Ala-OBzl

A total of 4.9 g (0.009 mol) of the peptide obtained in the previous step is dissolved in 50 mL of 80% trifluoroacetic acid (TFA) and stirred for 45 min. The reaction progress is monitored using TLC (system A). After the reaction, the solution is evaporated in a vacuum and precipitated with ether. Rf = 0.22 (A). The precipitate is filtered off and dried in air.

Yield: 4.5 g (92%).

TFA. H-D-Ala-Phe-Gly-OBzl (Rf = 0.8 (A)) l,

TFA. H-D-Ala-Gly-OBzl (Rf = 0.8 (A)) is obtained similarly.

7. The synthesis of Boc-Tyr-D-Ala-Phe-D-Ala-OBzl.

First, 0.6 g (0.003 mol) of Boc_2_-Tyr-OH is dissolved in 10 mL of dioxane, equimolar amounts of TBTU and NMM are added, the solution is stirred for 3 min. Then, a solution of 1.6 g (0.0031 mol) of TFA.H-D-Ala-Phe-D-Ala-OBzl in 15 mL of dioxane is added. The solution is stirred with a magnetic stirrer for 2 h, maintaining a pH of 7–8. The reaction progress is monitored using TLC (system A). After the reaction is complete, the solution is evaporated in vacuo. Next, 50 mL of EtOAc/H2O is added to the residue and transferred to a separatory funnel, and the pH is adjusted to 3. The organic layer is washed with 5% H_2_SO_4_, followed by H_2_O, then 5% NaHCO_3_, and finally H_2_OThe solution is dried over Na_2_SO_4_, then evaporated in vacuo. The oily residue is dissolved in 30 mL of ethyl acetate, and ether is added until an opalescence is achieved. The solution is left with the precipitate overnight at +8 °C in the refrigerator. The precipitate is filtered off, washed with ether and hexane, and then air-dried. Rf = 0.8 (A).

Yield: 1.8 g (95%).

Boc_2_–Tyr-D-Ala-Phe-Gly-OBzl (Rf =0,8, A) and Boc_2_-Tyr-D-Ala-Gly-OBzl

(Rf = 0.8, A) were obtained similarly.

8. The synthesis of Boc2-Tyr-D-Ala-Phe-D-Ala-Tyr-Pro-Ser(Bzl)-NH_2_.

A total of 0.6 g (0.003 mol) of Boc2-Tyr-D-Ala-Phe-D-Ala-OH is dissolved in 10 mL of dioxane, equimolar amounts of TBTU and NMM are added, and the solution is stirred for 3 min. Then, a solution of 1.6 g (0.0031 mol) of TFA.H-Tyr-Pro-Ser(Bzl)-NH2 in 15 mL of dioxane is added. the solution is stirred with a magnetic stirrer for 2 h, maintaining a pH of 7–8. The reaction progress is monitored with TLC (system A). After the reaction is complete, the solution is evaporated in vacuo. Next, 50 mL of EtOAc/H_2_O is added to the residue and transferred it to a separatory funnel, and the pH is adjusted to 3. The organic layer is washed with 5% H_2_SO4, followed by H_2_O, and then with 5% NaHCO_3_, followed by H_2_O. The solution is dried over Na_2_SO_4_ and then evaporated in a vacuum. The oily residue is dissolved in 30 mL of ethyl acetate, and then ether is added until opalescence is achieved. The solution with the residue is left at +8 °C overnight in the refrigerator. The precipitate is filtered off, washed with ether and hexane, and dried in air. Rf = 0.8 (A). Yield: 1.8 g (95%).

9. The synthesis of TFA. H-Tyr-D-Ala-Phe-D-Ala-Tyr-Pro-Ser(Bzl)-NHMe

First, 4.9 g (0.009 mol) of the peptide obtained in the previous step is dissolved in 50 mL of 80% TFA and stirred for 45 min. the reaction progress is monitored using TLC (system A). After the reaction, the solution is evaporated in vacuo and precipitated with ether. Rf = 0.22 (A). The precipitate is filtered and dried in the air. Yield 4.5 g (92%).

H-Tyr-D-Ala-Phe-Gly-Tyr-Pro-Ser(Bzl)-NH_2_ was obtained similarly.

Synthesis of Cyclo-[L-Lys(H-Tyr-D-Ala-Phe-D-Ala-Tyr-Pro-Ser)-L-Glu- (L-Trp-NH_2_)] (D3): First, 0.6 g (0.003 mol) of Boc_2_-Tyr-D-Ala-Phe-D-Ala-Tyr-Pro-Ser-OH is dissolved in 50 mL of DMF, equimolar amounts of TBTU and NMM are added, and the solution is stirred for 3 min. Then, a solution of 1.2 g (0.0031 mol) of TFA{Lys-Glu(Trp-NH_2_)}in 50 mL of DMF is added. The solution is stirred with a magnetic stirrer for 2 h, maintaining a pH of 7–8. HPLC is used to monitored the reaction progress. After the reaction is complete, the solution is evaporated in a vacuum. Next, 100 mL of EtOAc/H_2_O is added to the residue and transferred it to a separatory funnel, and the pH is adjusted to 3. The organic layer is washed with 5% H_2_SO_4_, followed by H_2_O, and then with 5% NaHCO_3_, followed by H_2_O. The solution is dried over Na_2_SO_4_ and then evaporated in a vacuum. The oily residue is dissolved in 30 mL of ethyl acetate, and then ether is added until opalescence is achieved. The solution with the residue is left at +8 °C overnight in the refrigerator. The precipitate is filtered off, washed with ether and hexane, and dried in air to yield 1.5 g (95%).

A total of 1.4 g (0.005 mol) of the peptide obtained in the previous step is dissolved in 50 mL of TFA and stirred for 45 min. The reaction progress is monitored using the HPLC system.

After the reaction, the solution was evaporated in a vacuum, and the oil was precipitated by adding ether. The residue was then filtered off, dried in air, and purified using preparative high-performance liquid chromatography (HPLC). The yield was 1.1 g (82%).
